# Somatic embryogenesis and plant regeneration of cassava (*Manihot esculenta* Crantz) landraces from Cameroon

**DOI:** 10.1186/s40064-015-1272-4

**Published:** 2015-09-04

**Authors:** Kone Mongomake, Oumar Doungous, Behnam Khatabi, Vincent N. Fondong

**Affiliations:** Department of Biological Sciences, Delaware State University, Dover, DE 19901 USA; Laboratory of Crop Breeding, Department of Natural Sciences, University Nangui Abrogoua, Abidjan, Côte D’Ivoire; Ekona Research Center, Institute of Agronomic Research for Development, Buea, South West Region Cameroon

**Keywords:** Cassava, Somatic embryogenesis, Organogenesis, Plant growth regulators

## Abstract

A procedure to regenerate cassava (*Manihot esculenta* Crantz) cultivars from Cameroon via somatic embryogenesis (SE) was developed. Shoot apical meristems and immature leaf lobes were used as explants on Murashige and Skoog (MS) basal medium containing 33 or 50 µM of the auxins Picloram (Pic), 2,4-Dichlorophenoxyacetic acid (2,4-D), Dicamba (Dic), and α-Naphthalene acetic acid. Cultivar performance was assessed using SE and number of somatic embryos produced. Overall, the frequency of primary somatic embryogenesis (PSE) and the mean number of somatic embryos produced varied considerably with genotype, type of auxin and concentration tested. For example, cultivar (cv.) Ngan Mbada showed the best performance on MS medium supplemented with 50 µM Pic with a SE frequency of 40 % and an average number of somatic embryos of 90. The second best performance was recorded in cv. Local Red on MS medium supplemented with 33 µM 2,4-D, where the SE frequency was 40 % and an average number of somatic embryos of 60.5. Cultivar Ekona Red recorded the best performance on medium supplemented with 50 µM Pic showing a SE frequency of 47 % and an average number of somatic embryos of 45. We further examined secondary and cyclic somatic embryogenesis (SSE, CSE) and both were also observed to vary with genotype, however, both exhibited significantly higher frequencies of SE compared with PSE. SE started to decline at the fourth cycle of embryogenesis. Examination of organogenesis showed that shoot bud induction from green cotyledons varied across cultivars and benzylaminopurine was shown to outperform Thidiazuron in the ability to induce organogenesis. Furthermore, the frequencies of bud induction were identical under light and dark conditions. Finally, regenerated plants grew easily in the greenhouse with 90–100 % survival rate and did not display detectable variation in morphology.

## Background

Cassava (*Manihot esculenta* Crantz) is a staple food to nearly a billion people in about 105 countries, providing as much as a third of daily calorie intake (FAO 2008a, b). World production was estimated at 250 million tons in 2011 (FAO 2012). In Africa, the continent with the largest production (53 % of world production), the crop plays an important role as famine-reserve crop, rural staple food, cash crop for both rural and urban households and, to a lesser extent, raw material for feed and chemical industries (Nweke et al. 2002). Because of its resilience and capacity to grow on marginal lands, it is predicted that the importance of cassava cultivation in farming systems affected by climate change will increase in the future (Lobell et al. 2008). Furthermore, cassava starch exhibits high purity, solubility, low tendency to retrograde compared with other starches such as potato, rice and corn; this makes cassava a promising source for biofuel production (Zamora et al. 2010).

Despite its potentials for achieving food security and economic growth, biotic and abiotic constraints such as diseases, pests, weeds, and drought are limiting cassava production (Barceloux 2009; Bull et al. 2011). In addition to these constraints, production has several other constraints, including toxic cyanogenic compounds, very low protein content (1–2 % dried weight) and short fresh tissue shelf life of 1–3 days (Westby 2002). To improve the crop, therefore, important traits have been introgressed through traditional breeding, leading to major improvements in resistance to bacterial blight and viruses (Okogbenin et al. 2007). Furthermore, advances have been made in improving protein content (Chávez et al. 2005) and starch quality (Ceballos et al. 2007) through breeding. However, traditional breeding techniques face several limitations, notably, high heterozygosity, allopolyploidy, low fertility, unsynchronized flowering and limited knowledge of inheritance traits that have agronomic importance (Nassar and Ortiz 2010). Thus, production of improved plant lines by conventional breeding is a long and tedious process (Ceballos et al. 2004; Rudi et al. 2010). Therefore, cassava genetic transformation has emerged as a valuable alternative and complementary approach to improve the crop (Sayre et al. 2011; Liu et al. 2011).

An important prerequisite to developing a genetic transformation system is the availability of morphogenic culture that can easily be used in gene transfer techniques (Taylor et al. 2004). In cassava, the most efficient procedure to producing morphogenic culture is through somatic embryogenesis, which has become an integral component of genetic transformation systems in cassava (Osorio et al. 2012). Regeneration studies have shown that the frequency and efficiency of somatic embryogenesis are genotype-dependent, and not all cassava cultivars are amenable to somatic embryogenesis, regeneration and/or transformation (Hankoua et al. 2005; Atehnkeng et al. 2006). This is yet an additional challenge to cassava improvement efforts. It therefore becomes necessary to optimize production of embryogenic structures for each cassava cultivar; yet, much of the research on cassava regeneration and transformation is currently largely devoted to varieties from South America (Taylor et al. 1993; Konan et al. 1994) even though much of the production is in Africa. Furthermore, previous reports showed that cassava cultivars from Africa respond differently in culture compared with South American varieties (Ihemere 2003). This study is aimed at investigating the ability of cassava genotypes from Cameroon to induce somatic embryos and regenerate plants via direct shoot regeneration from somatic cotyledons of maturing embryos. Results show that these Cameroonian cultivars are amenable to regeneration, even though the efficiency varied considerably with cultivar, auxin type and concentration. These results expand the range of African cassava cultivars that can be engineered using recombinant DNA technologies.

## Results

### Effect of plant growth regulators on induction of primary somatic embryogenesis

**Fig. 1:**
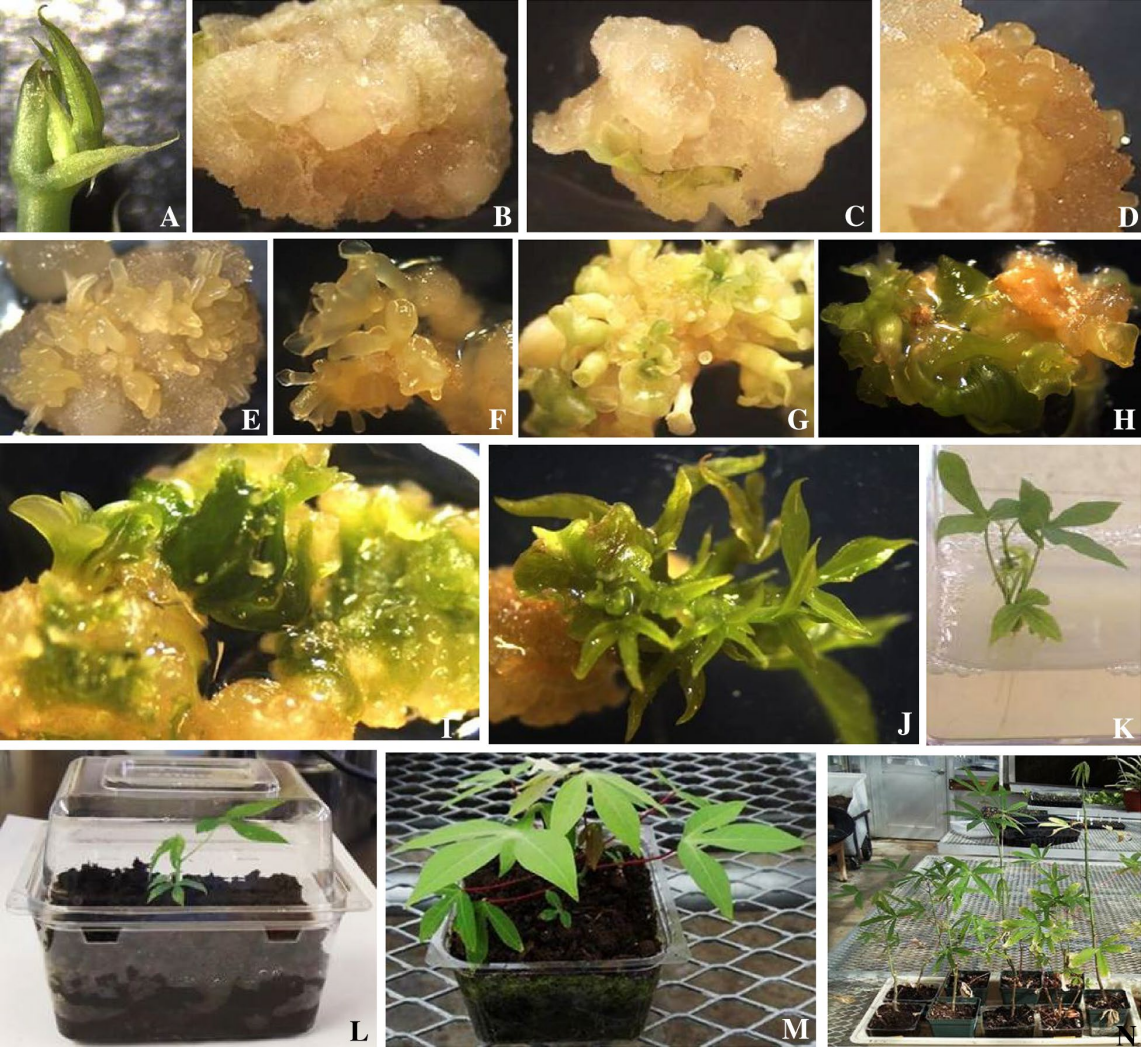
Regeneration of cassava cultivars from Cameroon. Apical immature meristematic leaf lobe explants (**A**) induced compact non-embryogenic callus (**B**) and callus with proembryogenic masses (**C**). Clusters of organized embryogenic structures consisting of globular (**D**) heart and torpedo structures (**E**), early cotyledonary stage (**F**), asynchronous development of somatic embryos (**G**) and green cotyledon (**H**) were observed. Organogenic callus with buds (**I**) derived from green cotyledons developed clusters of shoot buds (**J**). Elongated shoot buds rooted and developed into whole plantlets (**K**) in vitro. After transferring in boxes, hardened plantlets (**L**) were acclimatized (**M**) and they established in the greenhouse and grew into normal plants (**N**)

In this study, seven cassava cultivars from Cameroon were tested for their ability to induce primary somatic embryos on MS basal medium containing two concentrations (33 and 50 μM) of 2,4-D, Pic, NAA and Dic. The apical immature meristem leaf lobe explants (Fig. 1A) developed into a swollen callus mass on callus induction medium (CIM) within 5 days. From 3 to 4 weeks of culture, a compact non-embryogenic white callus (Fig. 1B) and translucent gelatinous callus with proembryogenic masses (Fig. 1C) were observed in all cultivars. These proembryogenic masses produced globular somatic embryos (Fig. 1D), which developed through the characteristic somatic embryogenesis stages of heart shape, torpedo and cotyledonary (Fig. 1E–H).

The potential of somatic embryogenesis, as indicated by the frequency of somatic embryo production, and the number of somatic embryos per explant, was assessed in each cultivar (Table 1). Results showed that both parameters varied widely across cultivar, auxin type and concentration. No primary somatic embryos were induced in medium containing 50 µM NAA and Dic, respectively (Table 1). Generally, the callus induced on NAA medium was soft and primary somatic embryos were not formed, instead, all explants formed abundant adventitious roots. Three cultivars produced no somatic embryos on medium supplemented with 33 µM Dic, while the other cultivars recorded a low frequency embryogenesis and few embryos were produced. The highest frequencies and number of somatic embryos per explant were observed in cv. Ngan Mbada (40.00 %; 90.00) on 50 µM Pic, followed by Local Red (40.00 %; 60.50) on 33 µM 2,4-D, and Ekona Red (46.67 %; 44.83) on 50 µM Pic (Table 1).

**Table 1:** Effect of plant growth regulators on somatic embryogenesis derived from immature shoot apical meristems of cassava cultivars from Cameroon

Plant growth regulators	Varieties	Frequency (%) of SE	Number of SE/explant
Picloram 33 µM	Sekelen	20.00 ± 5.77 e	08.00 ± 1.15 jk
	Ya Oroup	0.00 ± 0.00	0.00 ± 0.00
	Ngan Mbada	20.00 ± 5.77 e	59.33 ± 11.40 c
	Ekona White	0.00 ± 0.00	0.00 ± 0.00
	Local Red	60.00 ± 11.54 ab	08.00 ± 0.57 jk
	Ekona Red	0.00 ± 0.00	0.00 ± 0.00
	Local Ama	40.00 ± 11.54 cde	14.00 ± 3.21 ij
2,4-D 33 µM	Sekelen	40.00 ± 11.54 cde	21 ± 0.57 ghi
	Ya Oroup	0.00 ± 0.00	0.00 ± 0.00
	Ngan Mbada	0.00 ± 0.00	0.00 ± 0.00
	Ekona White	0.00 ± 0.00	0.00 ± 0.00
	Red Local	40.00 ± 5.77 cde	60.50 ± 3.50 c
	Ekona Red	40.00 ± 11.54 cde	26.50 ± 4.90 fg
	Local Ama	40.00 ± 5.77 cde	17.00 ± 3.46 hi
NAA 33 µM	Sekelen	0.00 ± 0.00	0.00 ± 0.00
	Ya Oroup	0.00 ± 0.00	0.00 ± 0.00
	Ngan Mbada	0.00 ± 0.00	0.00 ± 0.00
	Ekona White	0.00 ± 0.00	0.00 ± 0.00
	Local Red	0.00 ± 0.00	0.00 ± 0.00
	Ekona Red	0.00 ± 0.00	0.00 ± 0.00
	Local Ama	0.00 ± 0.00	0.00 ± 0.00
Diccamba 33 µM	Sekelen	20.00 ± 5.77 e	04.67 ± 0.67 k
	Ya Oroup	20.00 ± 11.54 e	04.67 ± 2.40 k
	Ngan Mbada	0.00 ± 0.00	0.00 ± 0.00
	Ekona White	0.00 ± 0.00	0.00 ± 0.00
	Local Red	20.00 ± 11.54 e	04.00 ± 2.08 k
	Ekona Red	0.00 ± 0.00	0.00 ± 0.00
	Local Ama	20.00 ± 5.77e	08.00 ± 2.08 jk
Picloram 50 µM	Sekelen	80.00 ± 11.54 a	44 ± 5.56 e
	Ya Oroup	0.00 ± 0.00	0.00 ± 0.00
	Ngan Mbada	40.00 ± 11.54 cde	90.00 ± 21.96 a
	Ekona White	63.33 ± 14.52 ab	32.00 ± 5.19 f
	Local Red	60.00 ± 20.00 ab	15.50 ± 3.32 ij
	Ekona Red	46.67 ± 17.63 bcd	44.83 ± 21.22 e
	Local Ama	40.00 ± 11.54 cde	47.66 ± 14.83 de
2,4-D 50 µM	Sekelen	40.00 ± 11.54 cde	21.00 ± 0.57 ghi
	Ya Oroup	20.00 ± 0.00 e	16.00 ± 2.08 ij
	Ngan Mbada	50.00 ± 5.77 bc	78.50 ± 2.02 b
	Ekona White	20.00 ± 0.00 e	34.00 ± 3.46 f
	Local Red	20.00 ± 0.00 e	22.00 ± 5.19 ghi
	Ekona Red	20.00 ± 0.00 e	54.00 ± 9.23 cd
	Local Ama	0.00 ± 0.00	0.00 ± 0.00
NAA 50 µM	Sekelen	0.00 ± 0.00	0.00 ± 0.00
	Ya Oroup	0.00 ± 0.00	0.00 ± 0.00
	Ngan Mbada	0.00 ± 0.00	0.00 ± 0.00
	Ekona White	0.00 ± 0.00	0.00 ± 0.00
	Local Red	0.00 ± 0.00	0.00 ± 0.00
	Ekona Red	0.00 ± 0.00	0.00 ± 0.00
	Local Ama	0.00 ± 0.00	0.00 ± 0.00
Diccamba 50 µM	Sekelen	0.00 ± 0.00	0.00 ± 0.00
	Ya Oroup	0.00 ± 0.00	0.00 ± 0.00
	Ngan Mbada	0.00 ± 0.00	0.00 ± 0.00
	Ekona White	0.00 ± 0.00	0.00 ± 0.00
	Local Red	0.00 ± 0.00	0.00 ± 0.00
	Ekona Red	0.00 ± 0.00	0.00 ± 0.00
	Local Ama	0.00 ± 0.00	0.00 ± 0.00

Within the same column, mean values followed by the same letter are not significantly different at α = 5 % (Newman–Keuls test)

±, standard deviation; SE, somatic embryos

The Pearson correlation coefficient was used to relate the frequency of somatic embryogenesis to average number of somatic embryos per explant. Except for cv. Local Red, a significant positive correlation was observed between both parameters, indicating that the higher the frequency of somatic embryogenesis the more somatic embryos are formed (Table 2).

**Table 2:** Correlations between frequency of somatic embryos induced and the average number of somatic embryos of cassava cultivars from Cameroon

Varieties	Correlations (% SE–NB SE)
Sekelen	0.848*
Ya Oroup	0.621*
Ngan Mbada	0.958*
Ekona White	0.719*
Local Red	0.382 NS
Ekona Red	0.818*
Local Ama	0.782*

*NS* non significant (bilateral test at α = 5 %)

* Significant

### Analysis of primary and secondary somatic embryogenesis on medium supplemented with Pic, 2,4-D and NAA

**Fig. 2:**
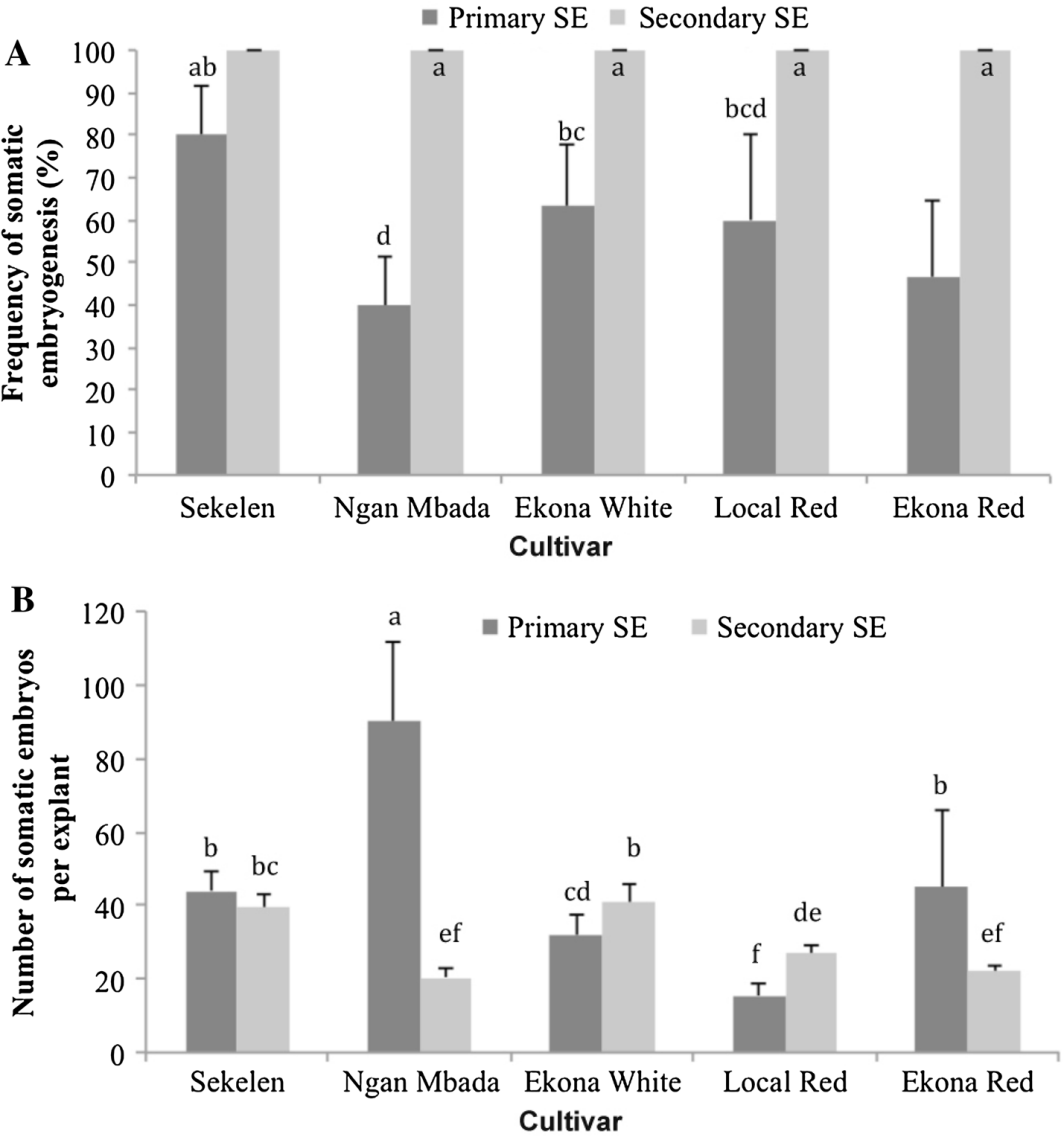
Efficiency of primary and secondary somatic embryogenesis as indicated by frequency of induction (**A**) and average number of somatic embryos per explant (**B**). Values represent the mean ± SD (*n* = 50). *Different letters* on *error bars* are significantly different at α = 0.05 (Newman–Keuls test)

We compared primary and secondary somatic embryogenesis on medium supplemented with 50 µM Pic and 2,4-D and NAA, since the concentration of 50 µM was found to be better than 33 µM in inducing embryo proliferation (Table 1). In this experiment, we used five cultivars (Sekelen, Ngan Mbada, Ekona White, Local Red, and Ekona Red). All five cultivars exhibited 100 % secondary somatic embryogenesis (Fig. 2A). However, cultivars behaved differently in the number of somatic embryos produced per explant (Fig. 2B). For example, cvs. Ngan Mbada and Ekona Red produced significantly higher average numbers of primary somatic embryos than the number of secondary embryos while cvs. Ekona White and Ekona Red produced significantly higher average numbers of secondary somatic embryos. As for cvs. Sekelen, there was no statistical difference between the numbers of primary and secondary embryos (Fig. 2B).

The effects of the three auxins on embryo induction frequency, number of somatic embryos per explant, and time (days) to complete the process, were determined. All three auxins induced secondary somatic embryogenesis in all five cultivars with frequencies varying from 57 to 100 % (Table 3). The frequencies of somatic embryogenesis were similar in cvs. Ekona White and Local Red under all three auxins. Pic and, to a lesser extent, 2,4-D induced a significantly higher frequency of somatic embryos than NAA in cvs. Sekelen, Ngan Mbada and Ekona Red. The lowest numbers of somatic embryos produced in all five cultivars were observed on medium supplemented with NAA (Table 3). The average number of days to complete somatic embryogenesis was significantly lower on NAA medium than on Pic or 2,4-D, both of which behaved similarly.

**Table 3:** Effect of auxins on secondary and cyclic somatic embryogenesis of five cassava cultivars from Cameroon

Varieties	Auxin (50 µM)	Frequency (%) of SE induction	Average number of SE per explant	Average days to complete the process
Sekelen	Pic	100.00 ± 0.00 a	56.25 ± 8.01 c	25.00 ± 2.22 a
	NAA	60.00 ± 0.00 d	22.00 ± 4.72 fg	11.00 ± 1.1 b
	2,4-D	73.33 ± 6.67 bc	55.67 ± 5.90 c	27.00 ± 3.5 a
Ngan Mbada	Pic	96.67 ± 3.33 a	37.20 ± 1.17 e	26.00 ± 3.7 a
	NAA	57.33 ± 1.33 d	11.33 ± 0.33 h	12.00 ± 1.5 b
	2,4-D	66.67 ± 6.67 cd	26.33 ± 2.60 f	24.00 ± 1.6 a
Ekona White	Pic	100.00 ± 0.00 a	92.33 ± 7.88 a	23.00 ± 2.3 a
	NAA	84.00 ± 2.08 ab	14.67 ± 2.72 gh	11.00 ± 1.6 b
	2,4-D	84.00 ± 2.08 ab	71.00 ± 11.23 b	22.00 ± 1.8 a
Local Red	Pic	100.00 ± 0.00 a	56.00 ± 4.93 c	28.00 ± 1.8 a
	NAA	100.00 ± 0.00 a	15.58 ± 1.52 g	12.00 ± 2.4 b
	2,4-D	100.00 ± 0.00 a	49.33 ± 8.11 cd	29.00 ± 0.8 a
Ekona Red	Pic	100.00 ± 0.00 a	44.00 ± 5.29 de	22.00 ± 4.2 a
	NAA	58.00 ± 2.00 d	11.00 ± 2.51 h	10.00 ± 0.6 b
	2,4-D	100.00 ± 0.00 a	46.67 ± 6.33 de	23.00 ± 4.5 a

Within the same column, mean values followed by the same letter are not significantly different at α = 5 % (Newman–Keuls test)

±, standard deviation; SE, somatic embryogenesis

### Effect of embryo cycling on plant regeneration

**Fig. 3:**
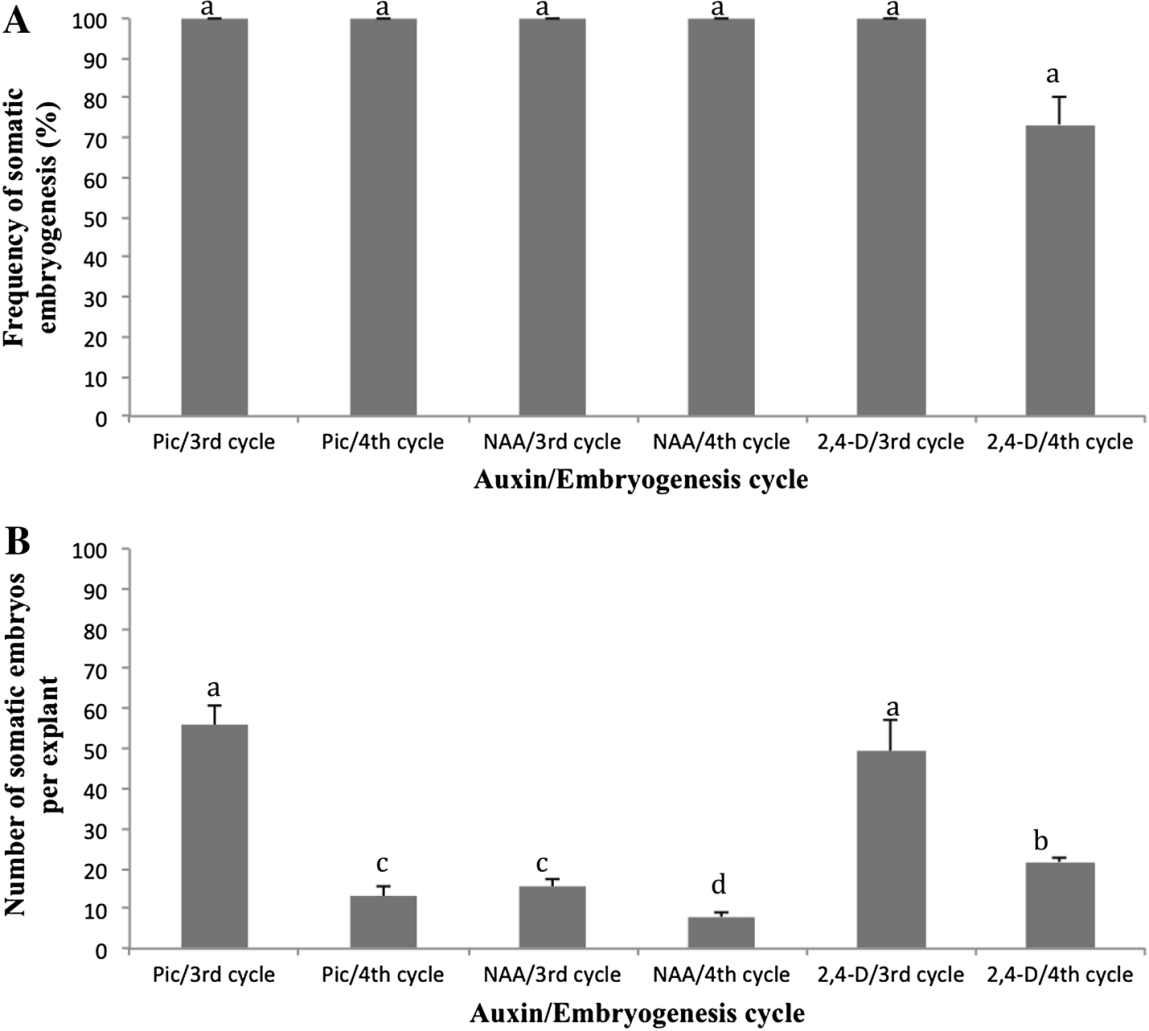
Influence of the number of cycles on somatic embryogenesis on medium supplemented with auxins Pic, NAA and 2,4-D as indicated by frequency of induction (**A**) and the average number of somatic embryos (**B**) were recorded. Data represent the mean ± SD (*n* = 50). *Different letters* on *error bars* are significantly different at α = 0.05 (Newman–Keuls test)

We investigated the effect of successive embryo cycling on induction of somatic embryos by determining the frequency and number of somatic embryos induced during the third and fourth cycles in the presence of Pic, 2,4-D and NAA. Results showed that on media supplemented with Pic and NAA, somatic embryo induction was observed to be 100 % in both third and fourth cycles (Fig. 3A). In contrast, a lower frequency was observed in medium supplemented with 2,4-D in the fourth cycle. As for the effect of cycling on the number of embryos, the third cycle showed a significantly higher number than the fourth cycle (Fig. 3B).

### Effect of BAP and TDZ on organogenesis under light and dark conditions

The effect of cytokinin BAP (1 mg/L) and cytokinin-like growth regulator, TDZ (0.022 mg/L), on organ production from green cotyledon somatic embryos was investigated under light and dark conditions. Frequencies of callus and bud formation as well as number of buds produced per explant are presented in Table 4. In medium supplemented with TDZ, no callus induction was observed under light or dark in all five cultivars. In medium supplemented with BAP, however, frequencies ranged from 81 to 100 % with no significant differences in the frequency of callus production under light and dark conditions across all five cultivars assessed.

**Table 4:** Effect of cytokinins and photoperiod on callus and bud induction from green somatic embryos of five cassava cultivars from Cameroon

Var	Cyt	Frequency (%) of callus induction		Frequency (%) of bud induction		Average number of buds per explant	
		Photoperiod	Dark	Photoperiod	Dark	Photoperiod	Dark
SK	BAP	91.00 ± 3.71 a	90.00 ± 3.33 a	69.00 ± 6.74 a	56.00 ± 7.18 a	13.48 ± 0.43 abcd	07.97 ± 1.20 cdef
	TDZ	0.00 ± 0.00 b	0.00 ± .00 b	12.00 ± 3.26 de	16.00 ± 5.81 cde	02.40 ± 0.70 e	0.90 ± 0.3 1 f
NM	BAP	81.00 ± 6.04 a	92.00 ± 4.42 a	53.00 ± 5.17 ab	66.00 ± 4.26 a	08.30 ± 0.72 ce	06.90 ± 0.30 def
	TDZ	0.00 ± 0.00 b	0.00 ± 0.00 b	0.00 ± .00 e	06.40 ± 2.97 e	0.00 ± .00 f	0.80 ± 0.29 ef
EW	BAP	81.77 ± 6.30 a	86.00 ± 3.05 a	71.55 ± 9.59 a	54.30 ± 7.38 ab	11.30 ± 0.74 bcde	06.48 ± 0.86 def
	TDZ	0.00 ± 0.00 b	0.00 ± 0.00 b	20.00 ± 0.00 cde	28.00 ± 7.42 cd	5.00 ± 0.00 ef	0.90 ± 0.23 ef
RL	BAP	100.00 ± 0.00	100.00 ± 0.00	56.00 ± 6.53 a	54.00 ± 5.81 ab	18.82 ± 1.52 ab	06.30 ± 0.65 def
	TDZ	0.00 ± 0.00 b	06.00 ± 3.05 b	10.00 ± 3.33 de	34.00 ± 7.91 bc	0.50 ± 0.16 f	02.45 ± 0.72 ef
ER	BAP	100.00 ± 0.00	100.00 ± 0.00	62.00 ± 5.53 a	62.70 ± 5.20 a	19.67 ± 2.70 a	15.26 ± 2.22 abc
	TDZ	0.00 ± 0.00 b	0.00 ± 0.00 b	0.00 ± .00 e	06.40 ± 2.97 e	0.00 ± .00 f	0.80 ± 0.29 ef

Within the same column, mean values followed by the same letter are not significantly different at α = 5 % (Newman–Keuls test)

±, standard deviation; Var, varieties; Cyt, cytokinins

As for shoot regeneration, all five cultivars produced shoots from secondary and cyclic embryos (Fig. 1i). Overall, the frequencies of bud formation were similar under light and dark conditions with higher values recorded in medium supplemented with BAP (53.00–71.55 %) than in medium containing TDZ (0.00–34.00 %) where the frequency of budding tended to be higher under dark (6.40–34.00 %) than under light (0.00–20.00 %) (Table 4). As for the number of buds formed, medium supplemented with BAP performed better than TDZ supplemented medium (Table 4). Taken together, organogenesis was higher in cvs. Ekona Red (62.72 %; 19.67 buds), Red Local (56.00 %; 18.82 buds) and Sekelen (69.00 %; 13.48 buds), while cv. Ngan Mbada recorded the lowest values (66.00 %; 8.30 buds) in medium supplemented with BAP.

### Rooting and acclimatization of regenerated plantlets

**Fig. 4:**
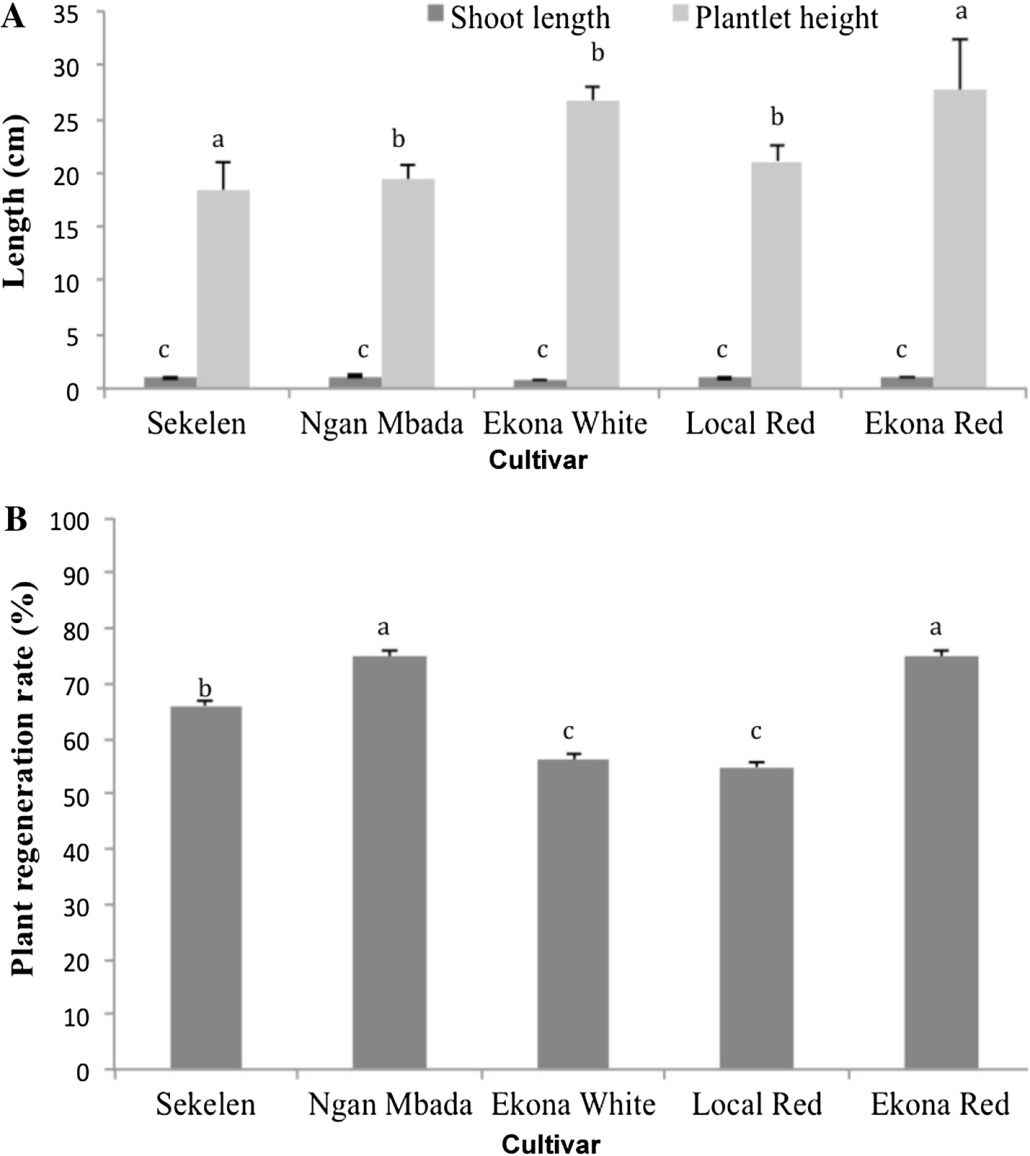
Shoot bud length on organogenesis medium, acclimatized plants height (**A**) and establishment of plants in the greenhouse (**B**). *Different letters* on *error bars* are significantly different at α = 0.05 (Newman–Keuls test)

Prior to transplanting to the greenhouse, lengths of shoots regenerated on maturation medium were measured; values ranged from 0.8 to 1.08 cm and showed no statistical differences (Fig. 4A). Shoots (Fig. 1J) of all cultivars developed roots efficiently on elongation medium supplemented with 0.4 mg/L BAP. We assessed the ability of regenerated plantlets to acclimatize and grow in the greenhouse by measuring the proportion of plantlets recovered as well as plantlet height. Cultivars Ngan Mbada and Ekona Red showed a significantly higher regeneration rate than cultivars Ekona White and Local Red (Fig. 4B). The regenerated plants were morphologically normal and grew rapidly (Fig. 1N) and after 6 weeks under greenhouse conditions, plantlets height ranged from 18 to 27 cm (Fig. 4A).

## Discussion

Despite its immense importance in the developing world, cassava has historically received less attention by researchers than have temperate crops (Olsen and Schaal 1999). Much of the genetic improvements of this crop have been through traditional breeding, which has resulted in the introgression into the cassava germplasm of bacterial and virus resistance (Hahn et al. 1980; Okogbenin et al. 2007) as well as other useful traits (Chávez et al. 2005; Ceballos et al. 2007; Morante et al. 2010; Rudi et al. 2010). Traditional breeding techniques face several limitations, notably the heterozygous nature of the crop, which renders it difficult to identify the true breeding value of parental lines. Furthermore, there is limited knowledge of inheritance traits that have agronomic importance (Ceballos et al. 2004; Olsen and Schaal 1999; Nassar and Ortiz 2010). These challenges, together with the fact that not all cultivated genotypes are amenable to breeding, not being able to produce flowers, make cassava improvement difficult. Thus, improvement through genetic engineering, which principally is carried out using *Agrobacterium*-mediated transformation of friable embryogenic callus (González et al. 1998; Zhang et al. 2001), has become a method of choice in cassava improvement.

Efficient *Agrobacterium*-mediated transformation of a recalcitrant crop as cassava depends on the ability to deliver intact DNA molecules into the genome of regenerable cells and to recover adult plants. Thus, production of somatic embryos, which are used as target tissue for insertion of T-DNA in the cassava genome, is a critical step in cassava transformation. In this study, we investigated the regeneration proficiency of cassava cultivars from Cameroon on media supplemented with Pic, Dic, NAA and 2,4-D. Visual assessment of culture using stereomicroscopy revealed the formation of somatic embryos exhibiting globular, heart shape, torpedo and cotyledonary stages on the same piece of callus tissue. This suggested that somatic embryogenesis is asynchronous, characterized by the presence of globular somatic embryos and embryos at more advanced stages of development on the same callus structure, similar to structures reported recently on cv. Cigana Preta, a Brazilian cassava cultivar (Vidal et al. 2014).

Addition of a strong auxin to the culture medium is known to efficiently induce in vitro somatic embryogenesis. Thus, 2,4-D was shown to be very efficient in inducing embryogenesis in *Anthurium andraeanum* (Pinheiro et al. 2013) and sweet potato [*Ipomoea batatas* (L.) Lam] (Magalhães et al. 2006). Here, we observed that all Cameroonian cassava cultivars were amenable to embryogenesis on media supplemented with Pic, 2,4-D and, to a lesser extent, Dic. In contrast, NAA failed to induce embryogenesis from leaf-lobe explants in all cultivars. These results were consistent with those of Sofiari et al. (1997), who assessed cultivars from Africa, South America and Asia. NAA tended to induce production of soft callus, which is not proficient in developing somatic embryos. Prolific induction of somatic embryos was obtained on media supplemented with 2,4-D and Pic. Both auxins have commonly been used in the induction of cassava somatic embryos (Li et al. 1996; Taylor et al. 2001; Zhang and Puonti-Kaerlas 2005). In our hands, Pic and 2.4-D at a concentration of 50 µM was more effective in inducing somatic embryos than 33 µM. Pic has constantly been shown to be more efficient in inducing embryo formation in African cassava cultivars (Ng and Adeniyi 1994; Raemakers et al. 1993; Rossin and Rey 2011), South America (Feitosa et al. 2007), and Asia (Li et al. 1998; Saelim et al. 2006). We also showed that the frequency of somatic embryogenesis and the number of embryos produced per explant varied with cultivar and auxin, suggesting a genotype-auxin interaction effect as reported by others (Feitosa et al. 2007; Saelim et al. 2006; Rossin and Rey 2011).

Somatic embryogenesis has consistently been assessed using induction frequency (Hankoua et al. 2005; Szabados et al. 1987) or number of somatic embryos per explant (Danso and Ford-Lloyd 2002; Feitosa et al. 2007; Ibrahim et al. 2008). These studies, however, have not investigated the possibility of a correlation between frequency and number of embryos formed. We have shown here that a significant positive correlation exists between frequency and number of somatic embryos produced in six of the seven cultivars investigated. This indicates that for most cassava cultivars, there is a high efficiency of proembryogenic mass conversion to various developmental stages of somatic embryos. It is therefore likely that these proembryogenic masses originate from cells that have acquired the capacity to induce formation of somatic embryos.

Consistent with previous reports on other cultivars (Hankoua et al. 2005; Sofiari et al. 1997; Zhang et al. 2001), we found that secondary somatic embryogenesis was induced in all five Cameroonian cultivars assessed in this study. However, we found that the frequency and number of somatic embryos varied across cultivars. The frequency of secondary embryogenesis was 100 % for all cultivars investigated, however, the number of embryos produced varied in primary and secondary embryogenesis, consistent with previous reports (Saelim et al. 2006; Sofiari et al. 1997). Furthermore, green secondary embryo explants were observed to produce more somatic embryos than isolated shoot apexes and would likely be excellent targets for genetic transformation. Cyclic embryogenesis appeared to affect the number of somatic embryos produced than the frequency. It is possible that loss of competence to convert proembryogenic masses to different developmental stages declines with successive embryo cycles, starting from the fourth cycle.

This study showed the inability of NAA to induce formation of primary somatic embryos from meristem leaf lobe explants even though it induced production of secondary somatic embryos from primary somatic embryos. This result is in agreement with results from others (Guohua and Qiusheng 2002; Sofiari et al. 1997; Raemakers et al. 1995). The efficiency of induction of secondary embryogenesis by 2,4-D, and especially NAA, varied considerably amongst all five cultivars assessed, thus, contrast with those of Sofiari et al. (1997), who reported that NAA was more proficient in inducing secondary embryogenesis than 2,4-D. We further found that NAA supplemented medium was more efficient at induction and maturation of secondary embryos compared with medium supplemented with Pic or 2,4-D. Indeed, production of green cotyledons from embryos took only 10 days on NAA medium whereas 3–4 weeks were required for Pic and 2,4-D. Thus, the whole embryogenic cycle using secondary somatic embryos can be completed in 14 days on NAA compared with at least 30 days for Pic or 2,4-D. Therefore, depending on the genotype, NAA is a plausible regeneration supplement since time is an important limiting factor in cassava regeneration.

Organogenesis from cotyledons of maturing somatic embryos is the most commonly used regeneration method for cassava (Fregene and Puonti-Kaerlas 2002). In contrast to the medium supplemented TDZ, callus induction was observed on the medium containing BAP. It is obvious that the auxin IBA combined with BAP might be responsible for this callus induction. Our results showed that BAP treatment gave the best organogenesis responses and thus in agreement with others (Guohua and Qiusheng 2002; Hankoua et al. 2005). It is not clear why TDZ was less efficient in inducing organogenesis from maturing somatic embryos, it is possible that the concentration may be an important factor and subsequent studies will need to assess different levels.

Although the frequency of bud induction was found in this study to be similar under light and dark conditions, the numbers of buds formed per explant were significantly higher when green cotyledons were incubated under 16 h light. The photoperiod has consistently been shown to be genotype-dependent for shoot formation. For example, a photoperiod of 16 h light was reported to be more efficient in inducing shoot formation from green cotyledons (Hankoua et al. 2005), while Li et al. (1998) obtained better results under continuous dark. We found that cv. Ngan Mbada was efficient in embryogenesis but less proficient in organogenesis, suggesting that the ability to produce somatic embryos does not necessarily translate to shoot regeneration proficiency. This result indicates that somatic embryogenesis and organogenesis may be controlled by different and independently inherited traits.

Taken together, this study shows that the Cameroon cultivars investigated here contain sufficient genetic variability for somatic embryogenesis and adventitious shoot formation and can likely be improved using the *Agrobacterium*-mediated approach. It is important to indicate that whereas some cassava cultivars from Colombia (Szabados et al. 1987; Mathews et al. 1993), Argentina (Medina et al. 2003) and Côte d’Ivoire (Konan et al. 1994) exhibit regeneration efficiencies similar to those reported here, others showed very low efficiencies.

## Conclusion

Factors that produced significant differences in T-DNA delivery and regeneration include plant genotype, explant source, embryo size, duration of pre-culture, inoculation and co-cultivation of *Agrobacterium*. The efficient whole plantlet regeneration protocol established here, allows for initiation of totipotent friable embryogenic callus, which is routinely used as target tissues for transgene insertion. Therefore, important traits such as resistance to cassava mosaic diseases, reduced toxic cyanogene content in tuberous roots, high protein content and drought tolerance can be introduced to these cultivars.

## Methods

### Plant materials

Seven farmer-preferred cassava cultivars from Cameroon were used in this study. Four of the cultivars are grown extensively in southwestern Cameroon, namely Ekona Red, Ekona White, Local Red, and Local Ama while three are grown in the north, Sekelen, Ya Oroup, Ngan Mbada. Plants were maintained by monthly subcultures of in vitro shoot cultures as described by Hankoua et al. (2005). The culture medium consisted of MS (Murashige and Skoog 1962) basal medium containing 20 g/L sucrose, 2 µM CuSO_4_ (pH 5.7), 0.8 % of Noble agar [cassava basal medium (CBM)]. The cultures were kept in a culture room at 25 ± 2 °C under 16 h light and light intensity of 3000 lx.

### Induction of somatic embryogenesis

Apical meristem-immature leaf lobes (AM-ILL) were excised from in vitro plantlets and cultured on MS basal medium containing 20 g/L sucrose, B5 vitamins (Gamborg et al. 1968), 2 μM additional copper in the form of CuSO_4_ (Schopke et al. 1992) and supplemented with 33 or 50 μM of auxins 2,4-Dichlorophenoxyacetic acid (2,4-D), Picloram (Pic), α-Naphthalene acetic acid (NAA), and Dicamba (Dic), respectively. Embryonic structures were examined using a stereomicroscope. Primary somatic embryos clusters containing globular, torpedo and heart-shaped structures were divided into units of 5–10 embryos. To develop green cotyledonary embryos or “maturing somatic embryos”, each cluster was transferred onto cassava maturation medium (CMML) (MS medium supplemented with 20 g/L sucrose, and 0.1 mg/L BAP) as described by Li et al. (1996).

To assess primary and secondary somatic embryogenesis, we used cultivars Sekelen, Ngan Mbada, Local White, Local Red and Ekona Red, which produced sufficient numbers of green cotyledonary somatic embryos for downstream experimentation. Green cotyledon pieces (5 mm^2^) were excised from the primary cotyledon embryos and transferred to P-CIM (callus induction medium supplemented with 50 µM Pic). Secondary somatic embryos were induced from green cotyledons of primary somatic embryos on 50 µM of Pic, NAA and 2,4-D, respectively. Induced secondary somatic embryos were then divided into small clusters of 5–10 and transferred onto CMML for maturation. Green cotyledon pieces obtained from 2 week-old secondary cotyledon embryos were placed on CIM supplemented with 50 µM Pic, NAA and 2,4-D for the induction of cyclic somatic embryogenesis.

Somatic embryogenesis was carried out in a growth chamber set at 25 ± 2 °C in continuous dark. Each treatment contained 10 Petri dishes and each Petri dish containing five explants (50 explants per treatment). The frequency of somatic embryogenesis and average number of somatic embryos produced at each stage per embryogenic callus were recorded from 3 to 4 weeks of culture.

### Effect of BAP and Thidiazuron (TDZ) on adventitious bud formation

Thidiazuron (TDZ) is an active cytokinin-like substance routinely used as a regulator, including stimulation adventitious shoot formation in woody plant tissue culture. The cytokinin, 6-benzylaminopurine, benzyl adenine (BAP) also elicits plant growth and development responses. Thus, we assessed the effect of both regulators on adventitious bud formation of the cassava cultivars after three and four cycles of somatic embryogenesis. To do this, somatic embryos were divided into clusters of 5–10 embryos, which were transferred to CMML for maturation. Matured green cotyledon embryos were then divided into 0.5 cm^2^ pieces and transferred on cassava organogenesis medium (COM) [MS basal medium, vitamins B5, 20 g/L sucrose and 2 µM CuSO_4_, supplemented with 1 mg/L BAP, 0.5 mg/L Indole butyric acid (IBA) or 0.022 mg/L TDZ, pH 5.7, and Noble agar (0.8 %)]. Each treatment contained 10 explants in each of five Petri dishes (50 explants per treatment). Cultures were incubated either in continuous dark or fewer than 16 h light to determine the effect of light on bud formation. After 1 month in culture, the frequency of callus and bud induction, the number of buds per explant and shoot bud length were recorded.

### Elongation and rooting of shoot buds, and acclimatization of regenerated plantlets

Shoot primordia from maturation medium were transferred onto cassava elongation medium (CEM: CBM supplemented with 0.4 mg/L BAP) for shoot elongation. After 4 weeks, the elongated shoots were transferred onto cassava rooting medium (CRM: CBM without plant growth regulators) for rooting and development. After root development, agar was rinsed from roots using tap water and the plantlets transferred to pots containing a Jiffy peat pellet. Pots were placed in closed transparent boxes to maintain high humidity and placed in the greenhouse where the temperature ranged from 18 to 25 °C and the relative humidity from 80 to 60 %. After 10 days, boxes were opened slightly to allow air circulation and 1 week later, the cover was completely removed. The percentage of plantlet survival and their heights were recorded 4 weeks after being transferred to the greenhouse.

### Experimental design and statistical analysis

All experiments were carried out in the completely randomized design. Samples were valuated using analysis of variance (ANOVA). Newman–Keuls multiple range tests were used to separate treatment means found significantly different by ANOVA. All analyses were at P ≤ 0.05 confidence level. Analysis was performed with the statistica 7.0 software.

## Abbreviations

2,4-D: 2,4-Dichlorophenoxyacetic acid
BAP: benzylaminopurine
CBM: cassava basal medium
CEM: cassava elongation medium
CIM: callus induction medium
CMML: cassava maturation medium
COM: cassava organogenesis medium
CRM: cassava rooting medium
CSE: cyclic somatic embryogenesis
NAA: α-Naphthalene acetic acid
P-CIM: callus induction medium supplemented with Pic
Pic: Picloram
PSE: primary somatic embryogenesis
SAM-ILL: shoot apical meristems immature leaf lobes
SE: somatic embryogenesis
SSE: secondary somatic embryogenesis
TDZ: Thidiazuron
